# Scaling of Soaring Seabirds and Implications for Flight Abilities of Giant Pterosaurs

**DOI:** 10.1371/journal.pone.0005400

**Published:** 2009-04-29

**Authors:** Katsufumi Sato, Kentaro Q. Sakamoto, Yutaka Watanuki, Akinori Takahashi, Nobuhiro Katsumata, Charles-André Bost, Henri Weimerskirch

**Affiliations:** 1 International Coastal Research Center, Ocean Research Institute, The University of Tokyo, Otsuchi, Iwate, Japan; 2 Graduate School of Veterinary Medicine, Hokkaido University, Sapporo, Japan; 3 Graduate School of Fisheries Sciences, Hokkaido University, Hakodate, Japan; 4 National Institute of Polar Research, Itabashi, Tokyo, Japan; 5 Centre d'Etudes Biologiques de Chizé-CNRS, Villier en Bois, Beauvoir/Niort, France; University of Hull, United Kingdom

## Abstract

The flight ability of animals is restricted by the scaling effects imposed by physical and physiological factors. In comparisons of the power available from muscle and the mechanical power required to fly, it is predicted that the margin between the powers should decrease with body size and that flying animals have a maximum body size. However, predicting the absolute value of this upper limit has proven difficult because wing morphology and flight styles varies among species. Albatrosses and petrels have long, narrow, aerodynamically efficient wings and are considered soaring birds. Here, using animal-borne accelerometers, we show that soaring seabirds have two modes of flapping frequencies under natural conditions: vigorous flapping during takeoff and sporadic flapping during cruising flight. In these species, high and low flapping frequencies were found to scale with body mass (*mass* ^−0.30^ and *mass* ^−0.18^) in a manner similar to the predictions from biomechanical flight models (*mass* ^−1/3^ and *mass* ^−1/6^). These scaling relationships predicted that the maximum limits on the body size of soaring animals are a body mass of 41 kg and a wingspan of 5.1 m. Albatross-like animals larger than the limit will not be able to flap fast enough to stay aloft under unfavourable wind conditions. Our result therefore casts doubt on the flying ability of large, extinct pterosaurs. The largest extant soarer, the wandering albatross, weighs about 12 kg, which might be a pragmatic limit to maintain a safety margin for sustainable flight and to survive in a variable environment.

## Introduction

Albatrosses fly thousands of kilometres in a few days to forage [1] and always return to their nesting grounds during breeding. When albatrosses are viewed from the deck of a ship, they seem to transit effortlessly with the ship for a prolonged period with no apparent flapping of their wings. A combination of the long, narrow, aerodynamically efficient wings and the anatomical capability to lock their wings in a stretched position [2] permits albatrosses to travel with the lowest energy expenditure among seabirds [3]. While albatrosses are highly specialised for soaring, this does not exactly mean that their flight consists only of gliding; rather, they have been observed flapping their wings under calm wind conditions [2]. According to heart beat rate measurements, the flight cost of wandering albatrosses is the highest during takeoff and is higher during flight in headwinds than when the wind is behind them [4]. One possible explanation is that, for albatrosses, both takeoff and flying into headwinds requires relatively more flapping.

Precise kinematic descriptions of wing flapping by free-flying birds are still rare in the literature [5]. In particular, measuring the quantitative characteristics of an entire flight under natural conditions, from takeoff to landing, has been virtually impossible. However, due to recent innovations in measuring technology, small accelerometers have been developed for the study of flight kinematics in the field. Using these animal-borne accelerometers, we continuously monitored the flight performance of albatrosses and petrels during their long-distance foraging trips at sea. Based on these data, scaling analyses were conducted for five procellariiform species, including streaked shearwater *Calonectris leucomelas* (mean body mass = 0.6 kg, *n* = 7), white-chinned petrel *Procellaria aequinoctialis* (1.3 kg, *n* = 5), sooty albatross *Phoebetria fusca* (2.3 kg, *n* = 2), black-browed albatross *Thalassarche melanophrys* (3.4 kg, *n* = 4) and wandering albatross *Diomedea exulans* (9.4 kg, *n* = 8), the largest soaring bird. The aim of this study was to investigate how these species flap their wings for sustainable flight under natural conditions and to predict an absolute value for the maximum body size of albatross-like soarers.

## Results

**Figure 1:**
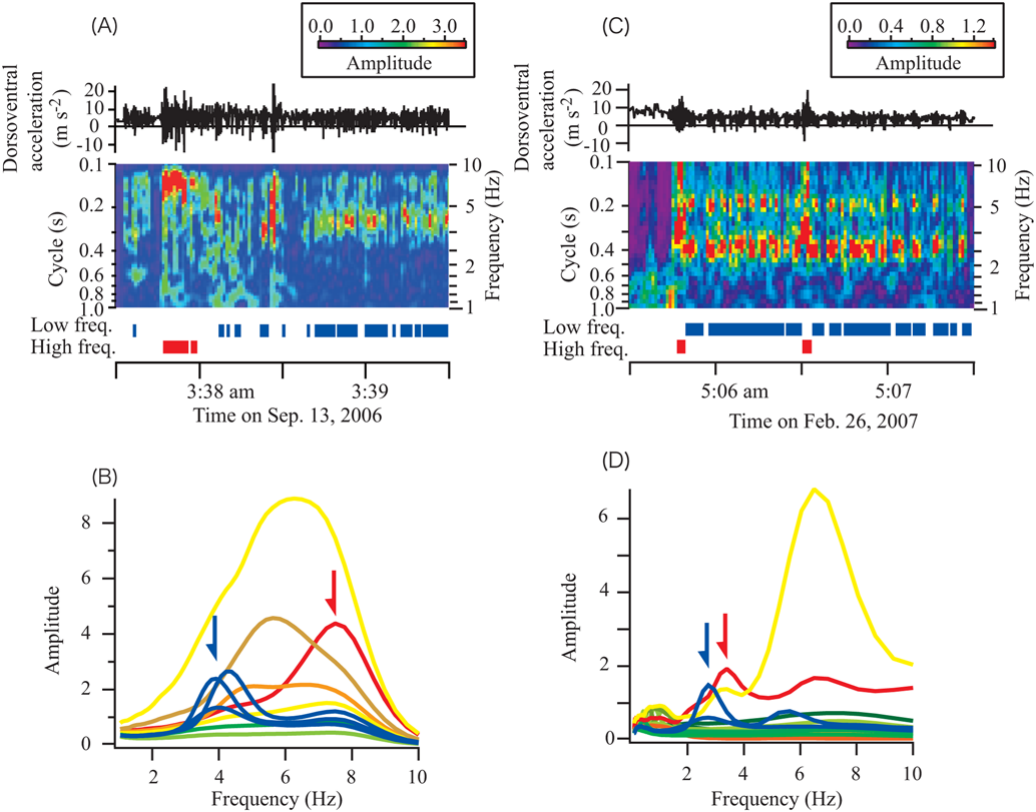
Spectrogram calculated from dorsoventral acceleration (black line) of a streaked shearwater and a wandering albatross during takeoff from the sea surface and subsequent flight. Ten discrete spectra were obtained from the entire data of the streaked shearwater (B) and 20 from the wandering albatross (D). Arrows indicate the frequencies regarded to be used for takeoff (red) and sporadic flapping (blue). Red and blue horizontal bars in (A) (a streaked shearwater) and (C) (a wandering albatross) indicate periods defined as high- and low-frequency flapping, respectively.

Figure 1A provides an example of an acceleration record for a streaked shearwater during takeoff from the water surface and subsequent flight. A spectrogram calculated from the time series data of acceleration indicated that the shearwater flapped with a high frequency (7.5 Hz) at the beginning and then sporadically, with a lower constant frequency (4.2 Hz), throughout cruising flight. Unsupervised cluster analysis *k*-means methods were used to obtain 10 discrete spectra from the entire data of this individual (Figure 1B). Two frequency ranges, 7.5 and 3.9–4.4 Hz (red and blue spectra in Figure 1B), were regarded to correspond with continuous flapping during takeoff and sporadic flapping during cruising flight, respectively. These frequency ranges were also appeared in other flight segments for this individual. Several spectra had strong amplitudes throughout the entire data set for this bird (yellow, orange and brown spectra in Figure 1B), however, these might correspond with other behaviours such as preening, foraging or digging holes, because most of these spectra occurred while the bird was on land, on the sea surface, and in the water. Spectra with weak amplitudes indicate that the source behaviour consists of weak movements or resting. Several gaps in the red and blue bars in Figure 1A indicate that the bird glided between wing flaps.

Data obtained from a wandering albatross, the largest soaring bird, indicate a similar pattern (Figure 1C). According to the spectrogram (Figure 1C), the bird flapped with a high frequency (3.4 Hz) at the beginning of flight and flapped sporadically with a low frequency (2.7 Hz) during flight. Twenty discrete spectra were obtained from the entire data set for this individual. We considered that frequencies of 3.4 and 2.7 Hz (red and blue spectra in Figure 1D, respectively) corresponded with flapping during flights. These frequencies were appeared in other flight segments for this individual. The difference between the high and low flapping frequencies of the wandering albatross was less than in the streaked shearwater (Figure 1B and 1D). An additional spectrum with a strong amplitude was obtained for this individual (yellow spectrum in Figure 1D). However, this spectrum occurred when the bird landed on the sea surface, which suggests that the spectrum corresponded with preening or foraging.

**Figure 2:**
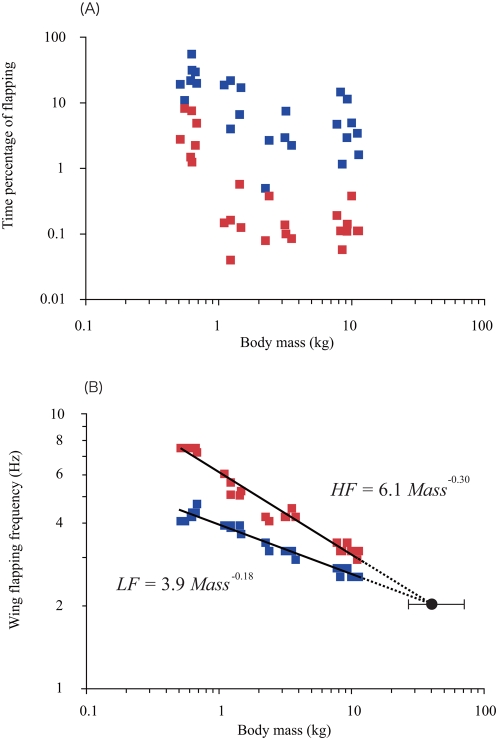
Body mass relationships. (A) The relationship between body mass and time percentage of slow (blue plots) and quick (red plots) flapping in a foraging trip of all individuals from the five species of albatrosses and petrels. (B) The relationship between body mass and wing-flapping frequencies. Regression lines were calculated for high (red plots) and low (blue plots) frequencies using MA estimation. Dashed lines were extrapolated for larger animals. The two lines intersect at a body mass of 41 kg (5.1-m wingspan), as indicated by a black circle and 95% CI (26–75 kg).

The same method of analysis was conducted for all individuals (see Figure S1, S2, S3). All birds (n = 26) in the five species of Procellariiformes had ‘top’ and ‘low’ gears for wing flapping. The percentage of time spent flapping varied among individuals within each species (Figure 2A). Larger-bodied species spent relatively lower percentages of their time performing slow flapping (Figure 2A), which indicates that they flapped less frequently. Based on our data, wandering albatrosses spent only 1.2–14.5% of their time performing slow flapping and 0.1–0.4% in quick flapping, i.e., not zero (Figure 2A). The relationships between flapping frequency and body mass for all birds (n = 26) are plotted in Figure 2B. Allometric equations were calculated for high and low flapping frequencies, respectively (Table 1). Both high and low flapping frequencies decrease, albeit with different slopes, according to the size of the bird (Figure 2B). The lower and higher stroke frequencies were proportional to *mass* ^−0.18^ and *mass* ^−0.30^. The scaling exponent of the lower frequency in relation to body mass was not significantly different from −1/6 (Table 1). However, the scaling exponent of the higher frequency was significantly different from −1/3 (Table 1).

**Table 1:** Allometric relationships between flapping frequencies (*HF*: high frequency in Hz, *LF*: low frequency in Hz) and body mass (*Mass* in kg) for 26 birds from 5 species and between wing sizes (*WS*: wing span in m, *WA*: wing area in m^2^) and body mass for 22 birds from 4 species.

Relationship	*γ*	95% CI for *γ*	*β*	95% CI for *β*	Predicted *β*	*P*
*HF* = *γ*×(*Mass*)*^β^*	6.1	5.9–6.4	−0.30	(−0.33)−(−0.27)	−1/3	0.01
*LF* = *γ*×(*Mass*)*^β^*	3.9	3.8–4.0	−0.18	(−0.20)−(−0.16)	−1/6	0.12
*WS* = *γ*×(*Mass*)*^β^*	1.3	1.2–1.3	0.37	0.34–0.40	1/3	0.03
*WA* = *γ*×(*Mass*)*^β^*	0.15	0.14–0.16	0.58	0.53–0.62	2/3	0.001

Scaling relationships have been calculated by major axis estimation for logarithmic values. Test statistics for the difference between observed and predicted values of slopes are given by *P*-values, taken from the *F*-distribution.

## Discussion

Albatrosses and petrels are generally recognized as soaring birds, which primarily rely on soaring during flight. However, the present study indicates that these species have two modes of flapping frequencies: vigorous flapping during takeoff and sporadic flapping during cruising flight. These flapping frequencies are necessary at certain stage of flight, when individuals conduct long distance migration during their foraging trips.

### High Flapping Frequencies for Takeoff

Since the introductions by David Attenborough in his book [6] and in his documentary film, streaked shearwaters have become famous as a seabird that climbs trees. Some ornithologists consider tree-climbing to be essential for takeoff in this species, compensating for the streaked shearwaters' limited capacity for flapping. However, these birds actually can take off from the ground by jumping into the air and vigorously flapping their wings (see Movie S1). Streaked shearwaters are pelagic seabirds that rely on marine food resources. During foraging trips at sea, individuals sometimes land on the sea surface and capture prey by surface-seizing or by performing shallow dives. The ability to achieve multiple takeoffs by wing flapping is therefore critical for the survival of streaked shearwaters. We found that individuals flapped their wings at higher frequencies, around 7.2–7.5 Hz, when taking off from the sea surface. The wandering albatross usually run on the ground or on the sea surface during takeoff (see Movie S2). When wandering albatrosses take off from the sea surface, they flap their wings at frequencies of 2.9–3.4 Hz, which is higher than the flapping frequencies observed during cruising flight (2.5–2.7 Hz). The percentage of time spent performing high-frequency flapping was not large (0.1–0.4%), however, it is essential for successful take off.

Takeoff is the transition from being supported by something that is essentially part of the earth's surface to being supported entirely by aerodynamic forces in flight, and these depend on air flowing over the wings [7]. Takeoff seems to be the most crucial task for flying birds and requires more active flapping than level flight because the flight speed is zero at the beginning and the birds must raise their body mainly by muscular effort. According to the heart rate measurements, effort was greatest when albatrosses took off [4]. Thus, we assumed that birds flapped their wings at the maximum power of their muscles when taking off. The upper limit of the flapping frequency would be proportional to *mass* ^−1/3^ for geometrically similar birds [8], [9], [10], [11]. Indeed, the observed scaling exponent (−0.30) of high flapping frequencies for takeoff was near the predicted value (−1/3). The present study compared phylogenetically similar species. The three species of albatrosses (Diomedeidae) and the two species of petrels (Procelariidae) belong to Procellariiformes. This order holds the most oceanic seabirds and span a huge size range from 20 g storm petrels to 12 kg albatrosses, which is a larger range than found in any other order of birds [12]. The long, narrow and aerodynamically efficient wings of these five species suggest larger capacity to migrate long distance. However, wingspan and wing area varied with the 0.37 and 0.58 powers of the mass, instead of the 1/3 and 2/3 powers as would be predicted on geometrical similarity (Table 1). The larger-bodied species had relatively longer and smaller wings as same as a previous study [2]. This might partially explain the discrepancy between observed and expected scaling coefficient for flapping frequency versus body mass.

### Low Flapping Frequencies for Sustainable Flight

In level flight, a bird must flap its wings to generate lift, and an optimum wing-flapping frequency exists at which lift and gravity forces on the bird are in equilibrium and mechanical power is minimum for sustainable flight performance [11]. Procellariiformes may be able to keep themselves airborne indefinitely without flapping their wings, if the surrounding air is moving [13], but when flight is not aided by the winds, the birds have to flap to avoid being pulled down by drag and gravity. In the present study, individuals sporadically flapped their wings and the percentage of time spent performing sporadic flapping varied among individuals within each species (Figure 2A), possibly because of variable wind conditions, as was reported in previous observations [2], [4]. The slow sporadic flapping of Procellariiformes during cruising flight is required to accelerate the birds' flight speed when wind conditions are unfavourable. The thrust (lift) produced by wing flapping is, 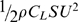, where *ρ* is the density of the air (kg m^−3^), *C*_L_ is the lift coefficient, *S* is the wing area (m^2^) and *U* is the speed of the wing (m s^−1^) [9], [10], [11], [14]. The wing speed *U* is proportional to the products of frequency *f* (Hz = s^−1^) and the amplitude *A* (m) of wing flapping: 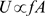. Assuming geometric and dynamic similarities (i.e., *C*_L_ = const., 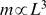, 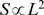 and 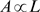, where *m* is the mass and *L* is the representative length of the body), thrust is proportional to 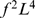. The amount of resistance that confronts a bird seeking to change its flight velocity can be quantified as a function of mass (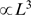). In other words, a bird with large body mass accelerate only with difficulty because of the large inertia. This situation can be expressed as 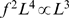. We thus obtain the following relationship for minimum flapping frequency with body mass for geometrically similar birds:
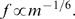
(1)This relationship is the same for continuously flapping birds [9], [10], [11], [14] and close to the obtained result of lower flapping frequencies proportional to *m* ^−0.18^ (Figure 2B, Table 1).

### Implication for the Maximum Size of Soaring Animals

Comparing the power available from muscles and the mechanical power required for flight, theoretical studies have predicted that the margin between these values should decrease with body size and that flying animals have a maximum body size [7], [10], [11], [13], [15], [16], [17]. Most of the previous studies assumed that mass-specific work and load lifting abilities are invariant with body size. However, predicting an absolute value for the upper limit on body size has proven difficult because wing morphology and flight style varies among species. Here we show that, in the Frequency-Mass diagram (Figure 2B), the two lines of the higher and lower flapping frequencies for phylogenetically similar species would, if extended, intersect at a body mass of 41 kg (5.1-m wingspan). The elevations and slopes of each allometric equation have confidence intervals (CI; Table 1) that affect the estimate of body mass at the intersection. The 95% CI for body mass at the intersection, which was calculated by bootstrapping (100,000 replicates), was 26–75 kg. Thus, albatross-like animals weighing close to 41 kg would lack any power margin to fly under unfavourable winds. Furthermore, an animal heavier than 41 kg would not be able to flap fast enough to increase its flight speed.

These deductions lead to an interesting implication regarding the maximum size of soaring animals, including extinct pterosaurs. Pterosaurs existed from the late Triassic to the end of the Cretaceous (220–65 million years ago) [18]. According to fossil-based estimates, their body mass ranged from 0.015 kg (0.4-m wingspan) to 70 kg (10.4-m wingspan). The morphology and flight ability of pterosaurs are widely debated [7], [17], [19], [20], [21]. Giant pterosaurs such as *Pteranodon* (16.6 kg, 6.95-m wingspan) and *Quetzalcoatlus* (70 kg, 10.4-m wingspan) are generally believed to have conducted soaring flight [18], [22], [23]. Other mass estimates of *Quetzalcoatlus* have ranged from 85 to 250 kg [24]. Based on our morphologic measurements of Procellariiformes (Table 1), for *Pteranodon* a body mass of 93 kg corresponds to a wingspan of 6.95 m while for *Quetzalcoatlus* a body mass of 276 kg corresponds to a wingspan of 10.4 m. If those large pterosaurs had extremely slender bodies, more so than albatrosses and petrels, the maximum power of their muscles would have been less and their flapping capacity accordingly diminished. Previous work on the flight performance of pterosaurs has often been based on the dogmatic assumption that pterosaurs were predominantly aerial piscivores living in coastal areas [24]. Partial skeletons of the largest pterosaur, *Quetzalcoatlus*, were discovered in continent 400 km from the nearest contemporary shoreline [20], [24]. It is suggested that *Quetzalcoatlus* might adopt vulture-like static soaring rather than dynamic soaring [24]. The present study does not deny the possibility that they might rely on warmed rising air of thermals using vulture-like broad wings. Precise flight performance of thermal soaring birds such as vultures, condors and frigate birds should be monitored under natural conditions.

Some studies have proposed that large pterosaurs such as *Pteranodon* and *Quetzalcoatlus* may have had narrow wings similar to those of albatrosses, and used slope soaring and dynamic soaring [18]. However, our study of living Procellariiformes as model animals suggests that if pterosaurs larger than 41 kg (or 5.1-m wingspan) had the narrow wings, they could not have attained sustainable flight in environments similar to the present. As demonstrated for extant albatrosses, which are mostly restricted to the Southern Ocean's “*roaring forties*”, where powerful winds blow consistently, flapping is necessary at certain stages of flight. Very specific environments, such as stronger and more constant winds, are essential for the sustainable flight of large pterosaurs. If other environmental factors (strength of gravity and density of the air) have changed over geological time, this might explain the brief appearance of large pterosaurs in the fossil record [7]. Alternatively, the results of the present study lend support to a recent reappraisal suggesting that large pterosaurs were terrestrial stalkers, finding much of their food via terrestrial, ground-level foraging [24]. Extant Procellariiformes employ the novel method of soaring to minimise the energetic costs of transit but they do not rely exclusively on soaring because the winds do not always allow it. Instead, these birds must have enough flapping ability to be able to take off from the sea surface and to attain sustainable flight under unfavourable winds.

## Materials and Methods

### Field Experiments

Field experiments were conducted under permission from the ethics committee of the Institut Polaire Paul Emile Victor, France, the Ministry of the Environment and the Agency for Cultural Affairs, government of Japan, and the Ethics Committee of the University of Tokyo. Data were obtained during breeding periods at Possession Island, Crozet Archipelago (wandering albatross, white-chinned petrels, sooty albatross in 2006/07), and the Kerguelen Islands (black-browed albatross in 2005/06), in the South Indian Ocean. Field studies in Japan were conducted on Sangan Island (streaked shearwater in 2006). Acceleration data loggers (D2GT, Little Leonardo Ltd., Tokyo, Japan) were used to detect the flapping movements of birds. The D2GT was 15 mm in diameter and 53 mm in length, with a mass of 18 g in the air; it recorded depth (1 Hz), two-dimensional acceleration (32 Hz) and temperature (1 Hz). The accelerometers were attached with waterproof tape to the feathers on the back or belly of the birds when departing for foraging trips and were retrieved when the birds returned to their nests. Body mass was measured using spring balances when data loggers were attached. Loggers were positioned to detect longitudinal and dorsoventral accelerations. The raw values recorded by the accelerometers were converted into acceleration (m s^−2^) as described previously [25].

### Data Analysis

To investigate modulation of the wing-flapping frequency throughout flying periods, a spectrogram of the dorsoventral acceleration was calculated by continuous wavelet transformation with the Morlet wavelet function [26], 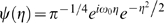, where *ω_0_* is the nondimensional frequency, here taken to be 10 to best differentiate the time and periodicity domains of the body acceleration. A spectrogram was calculated using the entire data set for each bird. Then, for each second, the spectrum was categorized into 10–50 discrete spectra using the *k*-means algorithm. *K*-means clustering is an unsupervised, interactive algorithm that minimizes the within-cluster sum of squared Euclidean distances from the cluster centroids. Each spectrum was defined by 64 values. Therefore, we performed *k*-means clustering in the same manner as clustering points in 64 dimensions [27]. Initially, we categorized each spectrum into one of 10 discrete spectra. If only one spectrum corresponded to flapping frequency, the number of discrete spectra was increased until flapping frequencies were categorized into two or more discrete spectra. Finally each flight period was composed of higher flapping frequencies during takeoff and lower sporadic flapping frequencies during cruising flight. The mean values of those frequencies were selected as representative high and low frequencies for flapping in each individual. The newly developed software “Ethographer” [28], which works on the Igor Pro (WaveMetrics, Inc., Lake Oswego, OR, USA) platform, readily allowed discrete stroke frequencies to be obtained from the spectrogram for each bird.

The main focus in the scaling analyses of the present study was on the slope of regression. Major axis (MA) estimations for the scaling relationships were performed in *R* [29]. Morphological measurements were conducted in the field when the data loggers were retrieved from birds (n = 22 in 4 species). Morphological data was not obtained from the black-browed albatrosses. As in a previous study [2], wingspan and wing area of each subject bird were measured including the torso segment between the wings. Scaling relationships were significantly different from one-third and two-thirds powers, as would be predicted based on geometric similarity (Table 1).

## Supporting Information

Figure S1 Spectrogram calculated from dorsoventral acceleration (black line) of a white-chinned petrel (A). Fifty discrete spectra were obtained from the entire data set for this bird (B). Arrows indicate the frequencies regarded to be used for takeoff (red) and sporadic flapping (blue). Red and blue horizontal bars in (A) indicate periods defined as high- and low-frequency flapping, repectively. The bird flapped with a high frequency (5.6 Hz) at the beginning of the flight, followed by low frequency flapping (3.9 Hz). This individual glided for a while and apparent flapping occurred two times during cruising flight (A).
(1.69 MB EPS)

Figure S2 Spectrogram calculated from dorsoventral acceleration (black line) of a sooty albatross (A). Fifty discrete spectra were obtained from the entire data set for this bird (B). Arrows indicate the frequencies regarded to be used for takeoff (red) and sporadic flapping (blue). Red and blue horizontal bars in (A) indicate periods defined as the high- and low-frequency flapping, respectively. In the case of the sooty albatross (n = 2), the signal corresponding to higher-frequency flapping was weak. However, the discrete spectrum indicated by a red arrow (B) was appeared at the beginning of flight.
(1.79 MB EPS)

Figure S3 Spectrogram calculated from dorsoventral acceleration (black line) of a black-blowed albatross (A). Fifty discrete spectra were obtained from the entire data set for this bird (B). Arrows indicate the frequencies regarded to be used for takeoff (red) and sporadic flapping (blue). Red and blue horizontal bars in (A) indicate periods defined as the high- and low-frequency flapping, respectively. Other spectra were apparent with this individual (B). Purple spectra (approximatly 2 Hz) corresponded with a periodical motion when the bird was on land. Orange and yellow spectra were obtained when the bird was on the sea surface.
(1.22 MB EPS)

Movie S1 It shows a streaked shearwater taking off from the ground by jumping into the air.
(3.11 MB MOV)

Movie S2 It shows a wandering albatross taking off from the ground by running on the hill.
(1.88 MB MOV)
